# 5th International Symposium on Synthesis and Catalysis (ISySyCat2023)

**DOI:** 10.3762/bjoc.20.227

**Published:** 2024-10-28

**Authors:** Anthony J Burke, Elisabete P Carreiro

**Affiliations:** 1 Faculty of Pharmacy, University of Coimbra, Pólo das Ciências da Saúde, Azinhaga de Santa Coimbra, 3000-548 Coimbra, Portugal; 2 Coimbra Chemistry Centre, Institute of Molecular Sciences, Chemistry Department, Faculty of Science and Technology, University of Coimbra, 3004-535 Coimbra, Portugal; 3 LAQV-REQUIMTE, Institute for Research and Advanced Training (IIFA), University of Évora, Rua Romão Ramalho, 59, 7000-671 Évora, Portugal

Organic synthesis and catalysis are two of the main stalwarts of the chemical sciences, and they have undergone extraordinary advances over the past 150 years. They are a crucial tool for the development of new molecules across a wide range of fields, including drug discovery, energy, materials science, and many more. The ability to design and create novel compounds through organic synthesis, aided by catalysis, is fundamental to advancing technologies that address global challenges in health, sustainability, and beyond. By enabling the production of complex molecules with specific functions, chemical synthesis and catalysis play a key role in innovation across the above-mentioned fields and thus improve our quality of life [1–10].

For almost 10 years, the International Symposium on Synthesis and Catalysis (ISySyCat) has brought together the biggest names in the fields of synthesis and catalysis, along with cohorts of dedicated practitioners, researchers, and students from these fields, presenting, discussing, and learning about the latest developments and cutting-edge innovations in the fields of synthesis and catalysis.

In this thematic issue dedicated to the 5th International Symposium on Synthesis and Catalysis (ISySyCat2023), which took place in Evora, Portugal from September 5–8, 2023, a diverse selection of contributions from a cross section of these participants is presented. The variety of material from different fields showcased in this thematic issue truly reflects the range and breath of this conference.

In the contribution by Dargó et al. [11], “A novel recyclable organocatalyst for the gram-scale enantioselective synthesis of (*S*)-baclofen”, an interesting approach to recycling the very useful cinchona squaramide organocatalysts was described. This approach involved functionalization of the organocatalyst with a lipophilic linker (octadecyl side chains), resulting in a novel lipophilic cinchona squaramide organocatalyst. This organocatalyst was evaluated in a benchmark Michael addition of acetylacetone to *trans*-β-nitrostyrene, yielding the Michael adduct with high yield and enantioselectivity. The hydrophobic chain of the catalyst allowed the organocatalyst to be easily recovered by precipitation using polar solvents. This catalyst proved to be excellent for the preparation of (*S*)-baclofen on a gram scale, furnishing the main chiral intermediate in high yield and enantioselectivity. Furthermore, the catalyst was recycled over five cycles while maintaining its performance.

In their contribution “Metal-catalyzed coupling/carbonylative cyclizations for accessing dibenzodiazepinones: an expedient route to clozapine and other drugs”, Moutayakine and Burke described a new synthetic route for the synthesis of 10,11-dihydro-5*H*-dibenzo[*b*,*e*][1,4]diazepinone (DBDAP) derivatives, which possess recognized pharmacological properties [12]. They used sequential reactions catalyzed by palladium and copper. The process involves an initial amination, which can be carried out via either the Buchwald–Hartwig or the Chan–Lam reaction, followed by a palladium-catalyzed intramolecular aminocarbonylation using Mo(CO)_6_. Both catalytic approaches successfully produced the desired DBDAPs.

As previously mentioned, organic synthesis is a crucial tool for preparing complex molecules of high value to industry. Frackenpohl et al*.* [13] designed and synthesized a new library of 2,3-dihydro[1,3]thiazolo[4,5-*b*]pyridine derivatives that exhibited strong herbicidal activity against commercially significant grass weeds in preemergence greenhouse tests. The synthetic route for this new family of compounds was developed and optimized, involving several reaction steps, included Pd-catalyzed Suzuki couplings and the reduction of the thiazole moiety to 2,3-dihydro[1,3]thiazolo[4,5-*b*]pyridines, a crucial intermediate, using BH_3_⋅NH_3_ and tris(pentafluorophenyl)borane as a Lewis acid, followed by treatment with formic acid.

Gillie et al. reported the synthesis of a laterally fused N-heterocyclic carbene (NHC) framework from polysubstituted aminoimidazo[5,1-*b*]oxazol-6-ium salts, which demonstrated strong catalytic activity in gold-catalyzed alkyne hydration and arylative cyclization reactions [14]. The synthesis of this new carbene involved the use of a novel nitrenoid reagent that was successfully synthesized on a gram scale through a three-step reaction sequence. The process began with 2,6-diisopropylphenylamine, which underwent alkylation, formylation, and substitution reactions. The carbene synthesis was then achieved via a two-step process involving ynamide annulation, followed by imidazolium ring formation. The resulting carbene was metalated at the C2 position with Au(I), Cu(I), and Ir(I), obtaining an L-shaped NHC ligand scaffold.

Līpiņš et al. introduced a new method for synthesizing 4-azido-6,7-dimethoxy-2-alkyl/arylsulfonylquinazolines, which are key intermediates in the preparation of biologically active compounds [15]. The synthesis was achieved via a sulfonyl group rearrangement driven by the azide–tetrazole equilibrium in quinazolines. The researchers utilized two synthetic pathways to prepare the target compounds. The first pathway involved a nucleophilic aromatic substitution (S_N_Ar) reaction between 2-chloro-6,7-dimethoxy-4-sulfonylquinazoline derivatives and NaN_3_, while the second involved an S_N_Ar reaction between 2,4-dichloro-6,7-dimethoxyquinazoline and alkyl/arylsulfinates, followed by substitution with NaN_3_. Using this developed methodology, the adrenoblockers terazosin and prazosin were successfully synthesized.

Oliveira Jr. et al. developed a new methodology for the asymmetric synthesis of β-aryl-γ-lactam derivatives with very good yield and enantioselectivity [16]. This was achieved through a palladium-catalyzed Heck–Matsuda desymmetrization of N-protected 2,5-dihydro-1*H*-pyrroles using aryldiazonium salts and (*S*)-PyraBox, followed by sequential Jones oxidation. They showcased their methodology by preparing both (*R*)-rolipram and (*R*)-baclofen hydrochloride.

Tóth et al. reported the design and synthesis of new analogues of HeE1-2Tyr, a nonnucleoside SARS-CoV-2 RdRp inhibitor, and their evaluation in an in vitro polymerase assay, targeting SARS-CoV-2 [17]. The synthesis of the new molecules involved three modifications of the HeE1-2Tyr inhibitor, which included changing the core structure from a benzothiazole to a benzoxazole unit and simplifying it to pyridone and thiazolopyridone derivatives. This work is interesting from the point of view that it involved the emerging technique of “chemical editing”. The pyridone and thiazolopyridone derivatives were the most promising inhibitors, with IC_50_ values below 90 µM.

Marques et al. described the synthesis of a new family of isatin-based α-acetamide carboxamide oxindole hybrids using the versatile Ugi four-component reaction [18]. Sixteen hybrids were prepared by reacting 5-amino-1-benzyl-3,3-dimethoxyindolin-2-one, benzyl isocyanide, carboxylic acids, and aldehyde/ketone derivatives, catalyzed by ZnF_2_ in MeOH at room temperature with a short reaction time. Some of them were further functionalized with a 1,2,3-triazole ring via copper-catalyzed azide–alkyne cycloaddition (CuAAC) and deprotected with trifluoroacetic acid. Several hybrids were evaluated against six cancer cell lines, displaying GI_50_ values in the range of 1–10 μM.

Teixeira et al. reported the preparation of new triazinephosphonate-based dopants and their application in the production of Nafion proton exchange membranes, which exhibited higher conductivity with only 1 wt % loading [19]. These new triazinephosphonate dopants could significantly impact the production of enhanced Nafion membranes, contributing to the development of more efficient decarbonized energy systems based on hydrogen technologies. The six triazinephosphonate derivatives bearing 4-aminophenyl or 4-hydroxyphenyl groups were obtained in very good yields through a nucleophilic substitution reaction between cyanuric chloride and 4-aminophenylphosphonate or 4-hydroxyphenylphosphonate derivatives. These synthesized dopants were used to prepare the modified Nafion membranes using a casting methodology.

Almodovar and Tomé reported the synthesis and characterization of nine novel diketopyrrolopyrrole derivatives through versatile S_N_Ar reactions between *N*,*N*’-bis(pentafluorobenzyl)-substituted diketopyrrolopyrrole and thiols and phenols under smooth conditions, resulting in the final compounds with satisfactory yields [20]. These newly synthesized compounds exhibited a high fluorescence quantum yield, which is an important property for their potential application in the field of optoelectronics (particularly for energy and biological chemistry applications).

In this thematic issue, Nieto et al. contributed a timely Review article on the chemical space of 2-phenethylamines, focusing on heteroaromatic structures with known pharmacological profiles [21]. They highlighted that changes in the phenyl and heteroaryl ring systems often stemmed from structure–activity relationship (SAR) exploration, where bioisosteric modifications of the original phenyl hits were prevalent. They reported that imidazole analogs behaved differently due to the ʟ-histidine unit representing a nonphenyl scaffold. Some important data on the bioisosteric modifications of 2-phenethylamine derivatives, focusing on their affinity and core aromatic diversity, were included.

In another Review article contributed to this thematic issue, Fehér et al. [22] carried out a critical assessment of the factors that affect the activity of immobilized organocatalysts. As mentioned earlier, organocatalysis has proven to be a powerful tool in the preparation of enantiopure compounds. However, their preparation can be time-consuming, complex, and expensive. Consequently, it is of utmost interest to immobilize them for reuse but without affecting their catalytic activity. The main factors discussed were the type of support, immobilization, and interaction between the support and the organocatalyst. The particular challenges were presented for each support, which are unique for different reaction substrates. Furthermore, the solutions to these problems as well as the limitations presented by these supports were discussed as well.

To conclude, you as a reader are encouraged to take a closer look at the contributions mentioned above, spanning diverse areas of modern synthesis and catalysis. We hope that you will find them beneficial, enlightening, and stimulating.

The 6th International Symposium on Synthesis and Catalysis (ISySyCat2025) will take place at the University of Coimbra from September 2–5, 2025 (https://isysycat2025.events.chemistry.pt/), and we hope that it will attract the attention of colleagues and practitioners from all corners of the world, who will share in the rich chemistry to be discussed during those four days.

As a final remark, we are very grateful to the *Beilstein Journal of Organic Chemistry* for the opportunity to publish a thematic issue dedicated to ISySyCat2023.

Anthony J. Burke and Elisabete P. Carreiro

Coimbra and Évora, October 2024

## Data Availability

Data sharing is not applicable as no new data was generated or analyzed in this study.
